# Hospital Relief in Holland

**Published:** 1911-01-07

**Authors:** 


### Editor's Letter-Box.

### HOSPITAL RELIEF IN HOLLAND.

*To the Editor of* The Hospital.

Dr. Van Eysselsteyn writes to us as follows:

With regard to the free days allowed (the 4,000 days granted to the medical staff), these are mainly intended for the Gynæcological Clinic. When an unmarried pregnant woman approaches the period of her delivery, the benevolent funds are usually not disposed to send her to the hospital to be treated at their cost. The argument is that she is, after all, not suffering from disease. And, in addition . . . ! On the other hand, married women are not disposed to permit themselves to be repeatedly examined by students. The Gynæcological Clinic, in a University hospital, can therefore only be carried on thoroughly, so far as education purposes are concerned, if patients are admitted free. At present the benevolent funds send us their gravidæ, while married women suffering from complications, such as contracted pelvis or repeated abortions, also enter the clinic. The 4,000 free days are mainly used for patients belonging to this division. But in the other departments the professors sometimes wish to admit special cases of patients who do not want to enter unless they can be admitted free of charge. The source of these interesting or, from an educational point of view, supremely useful, cases are the out-patient departments—the polyclinics. If one of the assistants of the out-patient department meets with an interesting case, which he thinks the professor would like to demonstrate to his class, he notifies the case to his chief. The patient is then given a letter to be delivered to the authorities of the municipality or congregation where he hails from. This letter simply states that his admission is desirable. If he now returns with an intimation that the authorities are prepared to pay for his admission, so much the better. On the other hand, if he returns and complains that the authorities are not disposed to pay for his treatment, he is admitted at the charge of the school. If he does not return at all, he is notified by letter that he will be admitted free of charge, but that his admission is urgently necessary in any case. Now and again it happens that a poor patient approaches us through his own (club) doctor, who writes to ask if So-and-so, with such-and-such symptoms, and tentatively such a diagnosis, can be admitted as a suitable patient for demonstration purposes. Then the professor sees the patient and judges for himself whether or not the latter's admission is desirable in the interests of the school, for, as I have already stated, the 4,000 days are mainly intended for the gynæcological department. Every year, however, the Minister of the Interior is requested to allow the professors an additional number of free days, but that number must never exceed 12,000 in all. The superintendent is responsible for the proper apportioning of these free days, and I make it a rule to require from the professors a quarterly return of the cases admitted gratis, and to see that this privilege is not abused. On page 11 of the annual report for 1909 you will find the statistical account of the number of free patients admitted. You will notice that there is a marked decrease during the long vacation (July to October). The other figures are of some importance for purposes of comparison. During the year we admitted 26,906 patients from Groningen itself, 24,835 from provincial municipalities, 7,522 from extra-provincial centres, 28,279 private (paying) patients, 2,562 patients from clubs, military, third-class paying patients generally, and finally 10,566 free patients. . . . The gynæcological department, I may mention, is on the corridor system, with wards for four to five patients. Married women may, if they desire, have a separate room, and those already delivered may also have, if they desire, a room apart from those not yet delivered. But there is one day room. There is thus scope for co-education and for womanly sympathy, which, on the whole, have been found very satisfactory.

With regard to the question of indigency. . . . The staff has no need to bother about the matter at all. If a patient is told that he ought to go to hospital the assistant at the polyclinic gives him a written statement to that effect. He must take this written statement to his own doctor—there are several club doctors in Groningen—or, if he has no doctor, then to his municipal medical authority—the poor doctor. This latter must decide whether the patient is to go to his proper pauper authority or to his club, and it is the duty of the officials of these organisations to find out what is the financial standing of the man or woman who applies for relief. These officials are responsible for the case, and are under the control of their own committees. If the patient is found to be really indigent, he is at once given a voucher to show that he will be treated at the expense of the club or organisation—pauper authority. If he is only relatively a pauper, that voucher is only given him after the pauper authority, in consultation with the committee, has definitely decided what sum he is to pay back to the committee after the latter has agreed to assist him. No patient is ever admitted to the hospital unless he is provided with a voucher or unless he has first deposited the required sum necessary for thirty days' treatment. In emergency cases—strangulated hernia, profound trauma, diphtheria, etc.—the superintendent, or, in his absence, the physician in charge, has the right of admitting a patient without requiring these guarantees; but in such cases the matter must be at once notified to the proper authorities, and I may mention that we rarely have any trouble in such cases. If payment is ultimately refused, we appeal to the Provincial Commission, and the patient is regarded as belonging to the educational department for the time being. Out-patients are examined free of charge, but are used for demonstration purposes; if they get a prescription they can have it made up at the hospital dispensary, but must pay for the medicine, unless they are provided with the necessary voucher already alluded to.

You can easily imagine that the indigent patients sometimes wish to make use of the benefits to be obtained from hospital treatment for a longer time than their cases warrant. Formerly we used to have regular chronics who came to spend the winter with us, all afflicted with that dreadful malady "Desiderium nosocomii"; which is very interesting, but not of much use for demonstration purposes! In order to deal with such cases, we are co-operating with the club and poor doctors, who visit such chronics, and, in consultation with the medical staff, treat them at home. We started this scheme two years ago, and it has so far worked excellently.

